# Occupational Hazard Among Biomedical Waste Handlers in Low‐Resource Settings: A Systematic Review and Meta‐Analysis

**DOI:** 10.1002/hsr2.72083

**Published:** 2026-03-16

**Authors:** Prakasini Satapathy, Abhay M. Gaidhane, Nasir Vadia, Soumya V. Menon, Kattela Chennakesavulu, Rajashree Panigrahi, Sanjit Sah, Kajal Samantaray, S. Govinda Rao, Khang Wen Goh, Rachana Mehta, Muhammed Shabil, Mahendra Singh, Edward Mawejje, Ganesh Bushi

**Affiliations:** ^1^ Center for Global Health Research, Saveetha Medical College and Hospital, Saveetha Institute of Medical and Technical Sciences Saveetha University Chennai Tamil Nadu India; ^2^ Jawaharlal Nehru Medical College, and Global Health Academy, School of Epidemiology and Public Health Datta Meghe Institute of Higher Education Wardha India; ^3^ Marwadi University Research Center, Department of Pharmaceutical Sciences, Faculty of Health Sciences Marwadi University Rajkot Gujarat India; ^4^ Department of Chemistry and Biochemistry, School of Sciences JAIN (Deemed to be University) Bangalore Karnataka India; ^5^ Department of Chemistry Sathyabama Institute of Science and Technology Chennai Tamil Nadu India; ^6^ Department of Microbiology, IMS and SUM Hospital Siksha ‘O’ Anusandhan (Deemed to be University) Bhubaneswar Odisha India; ^7^ Department of Paediatrics, Dr. D. Y. Patil Medical College Hospital and Research Centre Dr. D. Y. Patil Vidyapeeth (Deemed‐to‐be‐University), Pimpri Pune Maharashtra India; ^8^ Centre for Research Impact and Outcome, Chitkara University Institute of Engineering and Technology Chitkara University Rajpura Punjab India; ^9^ Department of Data Science Gokaraju Rangaraju Institute of Engineering and Technology, Bachupally Hyderabad Telangana India; ^10^ Faculty of Data Science and Information Technology INTI International University Nilai Malaysia; ^11^ Clinical Microbiology, RDC, Manav Rachna International Institute of Research and Studies Faridabad Haryana India; ^12^ University Center for Research and Development Chandigarh University Mohali Punjab India; ^13^ Department of Biotechnology Graphic Era (Deemed to be University) Clement Town Dehradun India; ^14^ School of Public Health Makerere University College of Health Sciences Mulago Hill Kampala Uganda; ^15^ School of Pharmaceutical Sciences Lovely Professional University Phagwara India

**Keywords:** biomedical waste, BMW training, hepatitis, low‐resource settings, meta‐analysis, occupational hazards, personal protective equipment, systematic review

## Abstract

**Background:**

Managing biomedical waste (BMW) effectively is a pressing public health challenge, particularly in low‐resource settings in accordance with the World Bank classification of low‐ and middle‐income countries (LMICs), where deficiencies in waste management infrastructure exacerbate health risks. BMW handlers face elevated occupational hazards due to inadequate safety measures, lack of training, and inconsistent use of personal protective equipment (PPE). Understanding these risks is crucial for improving health outcomes and ensuring the safety of these essential workers.

**Methods:**

This systematic review and meta‐analysis was conducted in accordance with PRISMA guidelines and registered with PROSPERO (CRD42024615074). We searched PubMed, Embase, and Scopus for peer‐reviewed articles published between January 2000 and October 2024, focusing on occupational hazards experienced by biomedical waste handlers in resource‐limited settings. Random‐effects models were used to estimate the pooled prevalence of needle and sharp injuries, hepatitis B and C infections, and the utilization rates of PPE. Heterogeneity among studies was quantified using the *I*² statistic, and sensitivity analyses were performed to ensure the robustness of our findings.

**Results:**

Fourteen studies met the inclusion criteria. The pooled prevalence of needle and sharp injuries was 26.4%. The prevalence of hepatitis B and C among BMW handlers was 5.8% and 2.4%, respectively. PPE usage rates were as follows: masks at 76.9%, gloves at 84.9%, boots at 19.0%, and gowns at 55.4%. Only 15.3% of handlers were immunized against hepatitis B, and 57.4% had received training in BMW management. Significant heterogeneity across studies indicates variable risk exposures and safety practices.

**Conclusion:**

The inconsistent use of PPE, along with low hepatitis B immunization and inadequate BMW training, highlights critical gaps in worker protection. Strengthening safety protocols, expanding training, and improving access to PPE are essential to reduce these risks. Given the substantial heterogeneity across studies, these findings should be interpreted with caution.

## Summary

- Biomedical waste handlers in low‐resource settings face significant health risks due to inadequate safety measures, with studies showing that 26% experienced needle and sharp injuries, and 5.8% and 2.4% were affected by hepatitis B and C, respectively.
- While some protective equipment like gloves (85%) and face masks (77%) are commonly used, essential items like boots and gowns are worn by fewer workers (19% and 55%).
- Additionally, only 15% of handlers are vaccinated against hepatitis B, and less than 60% have received proper training in waste management.
- These findings highlight the critical gaps in safety practices and the need for improved training, better access to personal protective equipment, and mandatory vaccinations to protect these workers from occupational hazards.

**Abbreviations**
ACIP: advisory committee on immunization practices
BMW: biomedical waste
BMWM: biomedical waste management
CDC: centers for disease control and prevention
CI: confidence interval
ES: effect size
GBD: global burden of disease
HCW: healthcare worker
HIV: human immunodeficiency virus
LFK: Luis Furuya–Kanamori index
MeSH: medical subject headings
NOS: Newcastle–Ottawa scale
NSI: needle stick injury
PPE: personal protective equipment
PRISMA: preferred reporting items for systematic reviews and meta‐analyses
WHO: World Health Organization

## 1 Introduction

Biomedical waste (BMW) management has become a critical global health and environmental concern, particularly in low‐resource settings in accordance with the World Bank classification of low‐ and middle‐income countries (LMICs) [1]. Inadequate waste disposal systems and occupational safety protocols exacerbate risks for biomedical waste handlers (BMW handlers) [2]. According to the World Health Organization (WHO), globally around 5.2 million people, including healthcare workers and waste handlers, are exposed to injuries or illnesses related to improper waste management each year [3]. With increased utilization of healthcare facilities and emergence of pandemics, the mountains of medical waste are growing exponentially [4]. Annually, 16 billion injections are administered worldwide, of which most are neither segregated nor disposed appropriately in developing countries [5].

BMW is any waste produced during the diagnosis, treatment, or immunization of human or animal research activities pertaining thereto or in the production or testing of biological or in health camps [6]. As per an estimate from the WHO, a low‐income country generates only 0.2–0.5 kg BMW per bed daily, whereas a developed country creates 0.5–3 kg BMW per bed daily [7]. Although low‐resource countries produce significantly less waste than developed countries, the lack of BMW management (BMWM) knowledge among healthcare workers and waste handlers, along with insufficient resources and technology, has substantially amplified the occupational hazard burden [8]. BMWM methods primarily aim at prevention, reduction, reuse, recycling, recovery, treatment, and ultimately disposal of BMW [5].

Biomedical waste handlers are individuals responsible for the collection, transportation, treatment, and disposal of BMW generated by healthcare facilities, laboratories, and other institutions dealing with biological materials. They are at higher risk of injuries related to needle sticks, cuts, and exposure to hazardous chemicals and infectious agents, which significantly increases the odds of acquiring bloodborne infections such as HIV, hepatitis B and C [9]. Therefore, WHO and CDC emphasize segregation of waste at the point of generation. However, in low‐resource settings, implementation is often inconsistent due to economic constraints and lack of awareness [10, 11].

Despite extensive research on occupational hazards within healthcare environments, there remains a noticeable deficiency in systematic reviews and meta‐analyses specifically addressing biomedical waste handlers, particularly in resource‐limited settings. This study aims to bridge this knowledge gap by focusing on the prevalence of occupation‐related hazards and safety measure utilization among biomedical waste handlers. By quantifying the adoption and implementation of these safety protocols, this research seeks to shed light on the effectiveness of current practices and identify crucial areas for improvement.

## 2 Methods

The PRISMA guidelines were followed in conducting this systematic review (Supporting Information S1: Table S1), and the review protocol was registered with PROSPERO (ID: CRD42024615074).

### 2.1 Selection Criteria

Studies were included if they reported primary data on occupational hazards or health outcomes related to handling biomedical waste, focused on populations in low‐resource settings in accordance with the World Bank classification of LMICs, and provided quantitative or qualitative data on health risks or injuries among biomedical waste handlers, published as full‐text articles in peer‐reviewed journals. Exclusion criteria were studies that were reviews, commentaries, letters, or conference abstracts without primary data; those focusing on biomedical waste handlers in high‐resource settings; and studies addressing general waste management practices without specific mention of occupational hazards for waste handlers.

### 2.2 Search Strategy

A comprehensive literature search was conducted to identify studies evaluating occupational hazards among biomedical waste handlers in low‐resource settings in accordance with the World Bank classification of LMICs. Databases including PubMed, Embase and Scopus were searched for relevant articles published between January 1, 2000 and October 1, 2024. The search terms included combinations of keywords and Medical Subject Headings (MeSH) terms, such as “biomedical waste handler,” “occupational hazards,” “resource‐limited settings,” “injury,” and “waste segregation,” The search was restricted to peer‐reviewed studies published in English, and references of included studies were manually screened to identify additional relevant articles (Supporting Information S1: Table S2).

### 2.3 Screening and Data Extraction

Screening and data extraction were conducted using Nested Knowledge software, which facilitated the removal of duplicates and streamlined the screening process. We employed a rigorous two‐step screening approach, first evaluating studies based on title and abstract, followed by a full‐text review to ensure they met our predefined eligibility criteria. This task was performed independently by two researchers, and discrepancies were resolved through discussion and consultation with a third researcher, ensuring an unbiased selection process.

Data extraction was conducted independently by two reviewers who gathered detailed information on study characteristics, participants, occupational hazards, use of personal protective equipment (PPE), and BMW training. Disagreements between the authors during the data extraction process were resolved through consensus with the third author.

### 2.4 Quality Assessment

The modified Newcastle–Ottawa Scale (NOS) was used to assess the quality of included studies [12]. Each study was independently assessed by the first and second reviewers based on study design, representativeness of the sample, and outcome measurement. Studies were categorized as high, moderate, or low quality, and only studies rated as moderate or high quality were included in the meta‐analysis. Any disagreements in quality ratings were resolved through discussion or by involving a third reviewer.

### 2.5 Data Synthesis and Statistical Analysis

Meta‐analysis was performed for studies with comparable data on the prevalence of injuries and health outcomes using R Studio (Version 4.4) metafor package. Random‐effects models were used to account for heterogeneity among studies. Heterogeneity was assessed using the *I*² statistic. Leave‐one‐out sensitivity analyses were conducted to examine the impact of study quality and regional differences on effect estimates. Doi plots and LFK values were used to assess publication bias.

## 3 Result

### 3.1 Literature Search

**Figure 1:**
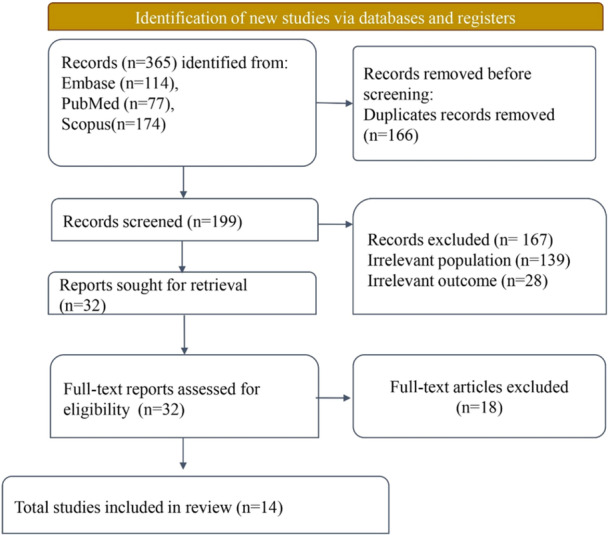
PRISMA flow diagram.

Initially, 365 records were retrieved from three databases. After removing 166 duplicates, 199 records remained for title and abstract screening. Of these, 167 studies did not meet the initial screening criteria, leaving 32 studies eligible for full‐text review. Following full‐text review of articles, 18 studies were further excluded, and finally, 14 studies were found to satisfy all inclusion criteria. The PRISMA flow diagram in Figure 1 outlines the systematic review process.

### 3.2 Study Characteristics

Fourteen studies were included for final analysis and data extraction in this systematic review and meta‐analysis (Table 1). Seven studies were conducted in Ethiopia [13, 14, 15, 16, 17, 18, 19], five in India [20, 21, 22, 23, 24], one in Libya [25], and one in Pakistan [9]. The majority of studies were set in tertiary government health facilities, and all employed a cross‐sectional study design. A total of 2713 biomedical waste handlers were recruited of which, approximately 32% male and 68% female. Participants' ages ranged from 18 to 60 years, with a mean age of around 35 ± 5 years. Most participants were either illiterate or had only elementary‐level education, while a smaller proportion had attained secondary education or higher. The Newcastle–Ottawa Scale for quality assessment (Supporting Information S1: Table S3) reveals that most included studies scored moderate to high quality (Supporting Information S1: Figure S1 depicting the summary of the quality assessment).

**Table 1:** Study characteristics of included studies.

Author, year	Country	Study design	Study setting	Sample size	Sex	Age	Qualification
Alemayehu et al. 2016 [13]	Ethiopia	Cross‐sectional survey	65 Public health facilities	253	Female—100%	29.7 ± 11.5 years	Literate—95.3% Illiterate—4.7%
Amsalu et al. 2016 [14]	Ethiopia	Cross‐sectional survey	Government hospital	152	Male—16 (10.5%) Female—136 (89.5%)	31.6 ± 8.3	Literate—121 (79.6%) Illiterate—31 (20.4%)
Anagaw et al. 2012 [15]	Ethiopia	Cross‐sectional survey	6 Government hospitals	100	Male—4 (4%) Female—96 (96%)	18–60	Literate—98 (98%) Illiterate—2 (2%)
Ayele et al. 2023 [16]	Ethiopia	Cross‐sectional survey	Healthcare	70	Male—15 (21.4) Female—55 (78.6)	18–>40	Illiterate 14 (20.0) Secondary level 20 (28.6)
Bhatia et al. 2018 [20]	India	Cross‐sectional survey	Healthcare	200	NA	NA	NA
Dandotiya et al. 2018 [21]	India	Cross‐sectional survey	Government hospital	87	NA	25	NA
Das et al. 2024 [9]	Pakistan	Cross‐sectional survey	Public and private tertiary hospitals	417	Male—297 (71.1%) Female 121 (28.9%)	18–50	Illiterate—200 (47.8%) Literate—218 (52.2%)
Franka et al. 2009 [25]	Libya	Cross‐sectional survey	Municipal medical management	300	Male—241 Female—59	NA	NA
Mahamed et al. 2024 [17]	Ethiopia	Cross‐sectional survey	Public hospital	417	Female—100%	< 25–> 35	Illiterate—251 (62.6%) Literate—166 (37.4%)
Mengiste et al. 2021 [18]	Ethiopia	Cross‐sectional survey	Healthcare	260	Male—8.8 Female—91.2	Median—29, IQR‐8	Illiterate—8.1%, Literate—91.9%
Shiferaw et al. 2011 [19]	Ethiopia	Cross‐sectional survey	Healthcare	126	Female—86 (68.3), Male—40 (31.7)	35.7 ± 9.7	Primary or less—58 (46%) Secondary— 68 (54%)
Shivalli et al. 2015 [22]	India	Cross‐sectional survey	Tertiary hospital	43	Female—100%	43.9 ± 6.5	Literate—30 (69.8%) Illiterate—13 (30.2%)
Singh et al. 2017 [23]	India	Cross‐sectional survey	Tertiary care hospital of Delhi	199	Male—81, Female—19%	30.6 ± 8.05	NA
Thakur et al. 2015 [24]	India	Cross‐sectional survey	11 Government hospitals	89	Male—50.6% Female—49.4%	38.5 ± 7.85	Illiterate—24.7% Literate—75.3%

### 3.3 Statistical Analysis

#### 3.3.1 Occupational Hazards Among BMW Handlers

Study findings show that occupational hazards among BMW handlers are alarmingly high. The prevalence of needle and sharp injuries among them varies significantly across studies, with the highest reported incidence being 71.1% and the lowest at 4.2% [17, 24]. Similarly, hepatitis B and C prevalence ranged between 2.3%–20.3% and 0.7%–4.3%, respectively [14, 16, 18, 25]. In terms of PPE, 57.7‐9% of BMW handlers use disposable gloves [20, 23, 25], 9‐67.5% use puncture‐proof boots [14, 19], use of face mask ranges between 17.7%–98% [20, 23, 25], and use of protective gown was 30%–90% [13, 18]. One study from India reported that the BMW handlers are not supplied with any kind of PPE kits [21].

### 3.4 Meta‐Analysis

#### 3.4.1 Prevalence of Needle and Sharp Injuries Among BMW Handlers

**Figure 2:**
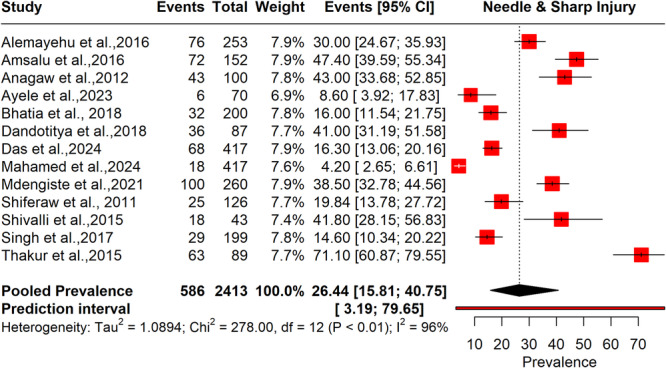
Pooled prevalence of needle and sharp injury among BMW handlers.

The meta‐analysis in Figure 2 reveals a pooled prevalence of needlestick and sharp injuries is 26.4% (95% CI: 15.8–40.7), indicating a notable risk for these workers. The heterogeneity among the studies is very high (*I*² = 96%), suggesting substantial variability in the injury rates reported across different settings and study designs.

#### 3.4.2 Prevalence of Hepatitis B and C Among BMW Handlers

**Figure 3:**
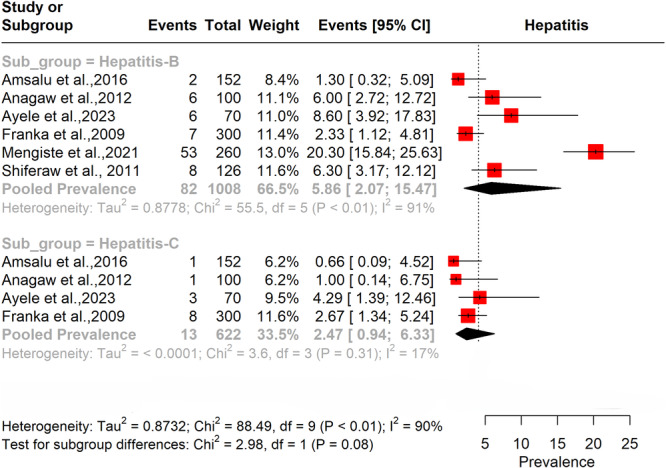
Pooled prevalence of Hepatitis B and C among BMW handlers.

The forest plot in Figure 3 shows that the prevalence of Hepatitis B among biomedical waste handlers is significantly higher than that of Hepatitis C. The pooled prevalence for Hepatitis B stands at 5.8% (95% CI: 2–15.4) with very high heterogeneity (*I*² = 91%), while Hepatitis C shows a lower pooled prevalence of 2.4% (95% CI: 0.9–6.3) with relatively low heterogeneity (*I*² = 17%).

#### 3.4.3 Prevalence of Use of Safety Measures by the BMW Handlers

**Figure 4:**
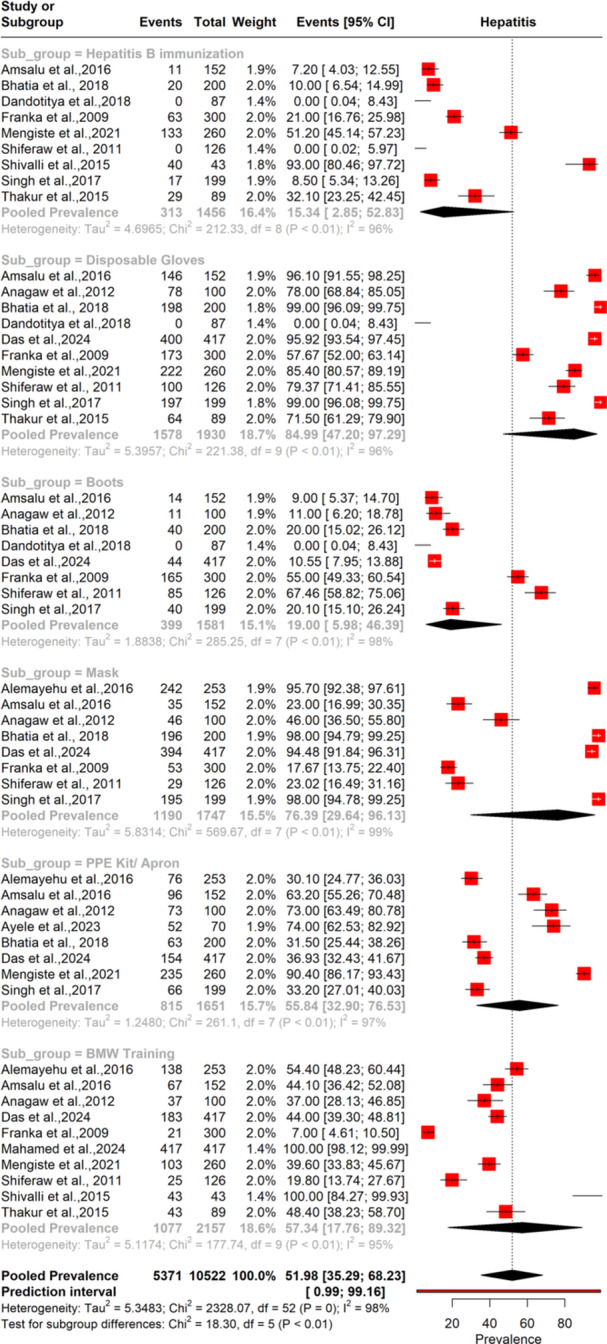
Pooled prevalence of use of safety measures by the BMW handlers.

The forest plot in Figure 4 highlights the prevalence of use of BMWM safety measures among BMW handlers. The pooled prevalence of Hepatitis B immunization among them is 15.3% (95% CI: 2.8–52.8), with significantly high heterogeneity (*I*² = 96%). However, the pooled prevalence of use of disposable gloves by them is 84.9% (95% CI: 47.2–97.2), with heterogeneity remaining high (*I*² = 96%). Boots are less commonly used by the BMW handlers, with a prevalence of 19.0% (95% CI: 15.9–46.3) with a heterogeneity of 98%. The pooled prevalence of mask use was moderately high at 76.9% (95% CI: 69.9–83.0) with the highest heterogeneity (*I*² = 99%). PPE kits or aprons are used by 55.4% of workers (95% CI: 43.5–66.8), with similarly high heterogeneity (*I*² = 99%). Lastly, BMW training is reported at 57.4% (95% CI: 48.0–66.5), with a heterogeneity of 95%.

### 3.5 Publication Bias

**Figure 5:**
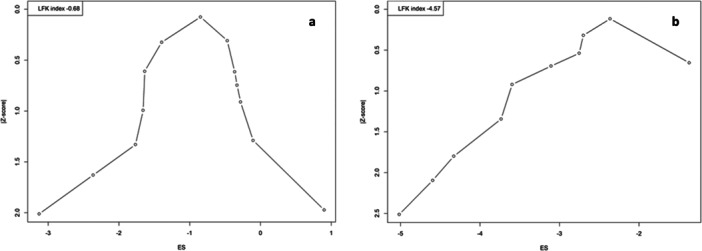
a and b: Doi plot representing the publication bias of injury and safety outcomes.

The needle and sharp injury doi plot (Figure 5a) shows moderate asymmetry, with an LFK index of −0.6. In contrast, the hepatitis doi plot (Figure 5b) displays marked asymmetry, with an LFK index of −4.5, indicating substantial bias.

### 3.6 Sensitivity Analysis

The sensitivity analysis for needle and sharp injury (Supporting Information S1: Figure S2) and hepatitis risk (Supporting Information S1: Figure S3) demonstrates the robustness of the meta‐analyses, showing that the pooled prevalence estimates remain consistent despite the individual removal of each study. The needle and sharp injury analysis reveals a pooled prevalence of around 0.2 (95% CI: 0.1–0.4), with consistently high heterogeneity at 96%. Similarly, in hepatitis, the pooled prevalence stabilizes around 0.04 (95% CI: 0.01–0.08), with *I*² = 90%, suggesting significant variability among studies. These findings indicate that the meta‐analytic conclusions are resilient and not overly dependent on any single study.

## 4 Discussion

Our review highlights significant occupational hazards faced by BMW handlers in low‐resource settings. Although subgroup analyses by region and publication year provided some insights, the limited number of studies within these groups restricts the strength of region‐specific conclusions. For instance, while some South Asian studies reported higher rates of needle and sharp injuries compared to those from Sub‐Saharan Africa, these differences should be interpreted with caution given small sample sizes and variability in study designs. Similarly, variations in the use of PPE and hepatitis prevalence across countries may reflect differences in healthcare infrastructure and reporting practices, but the available evidence remains too limited to support broad generalizations. Overall, we found a pooled prevalence of needle and sharp injuries among BMW handlers of 26.4%, which is lower than estimates for broader healthcare worker populations Bouya et al. reported a global 1‐year prevalence of 44.5%, and Mengistu et al. reported 32.4% [26, 27]. This discrepancy may be partly explained by underreporting among BMW handlers, who often lack adequate training and awareness of needlestick injury risks and reporting protocols compared to healthcare workers [23, 28]. Supporting this, a study from India demonstrated that awareness of biomedical waste management practices was highest among nurses (74.0%), followed by doctors (70.2%), while BMW handlers showed considerably lower awareness (53.8%) [29]. Taken together, these findings underscore both the heightened vulnerability of waste handlers and the need for cautious interpretation of regional or subgroup differences, emphasizing pooled estimates as more reliable indicators of occupational risks in low‐resource contexts.

Similarly, the pooled prevalence of seroprevalance of hepatitis B and C infection among BMW handlers were 5.8% and 2.4%. According to the Global Burden of Disease (GBD) estimate all‐age chronic Hepatitis B prevalence is 4.1% [30]. This highlights the disproportionate health risk within biomedical waste management contexts, as indicated by the significantly higher prevalence noted in our review [31]. This elevated prevalence is consistent with increased occupational risks such as sharp injuries and needle pricks, which are commonly reported incidents among waste handlers [32]. These injuries facilitate direct pathways for the transmission of bloodborne pathogens, significantly increasing the risk for Hepatitis B and C infections among this workforce [32].

Our study underscores significant violations of biomedical waste management (BMWM) protocols by healthcare organizations and waste handlers. According to the Advisory Committee on Immunization Practices (ACIP), mandatory hepatitis B vaccinations are crucial for the healthcare workforce, including waste handlers, to mitigate the risk of infectious diseases [33]. Nonetheless, our findings reveal a concerning scenario where only 15.43% of biomedical waste handlers are immunized against hepatitis B, indicating a serious gap in adherence to safety guidelines. Additionally, the use of other essential PPE is alarmingly low. For instance, only 19% of waste handlers use puncture‐proof boots, and just over half (55.4%) wear protective gowns. These findings reflect major gaps in ensuring the safety of those who handle potentially hazardous biomedical waste. Furthermore, while a moderate percentage of 57.4% of waste handlers have received some form of BMWM training, this indicates that nearly half of the workforce is operating without adequate knowledge of proper waste management practices, which is essential for both worker safety and environmental health. On a positive note, a higher percentage of waste handlers are using face masks (76.9%) and gloves (84.99%). This uptake in mask and glove usage is encouraging and suggests a level of awareness about personal health protection.

The critical disparity in the adoption of safety practices, essential for mitigating occupational disease risks, can be attributed to economic constraints that hinder the provision of necessary equipment and training, cultural and educational barriers that lead to non‐compliance with safety protocols, and inconsistencies in regulatory enforcement [34, 35]. These issues are particularly pronounced in developing countries, where rudimentary waste management systems and lack of regulatory oversight pose additional risks [36, 37]. Therefore, strengthening regulatory frameworks is essential to ensure consistent safety practices across regions [38, 39]. This includes enforcing the mandatory provision of PPE, regular health screenings, and compulsory hepatitis vaccinations [40]. Training programs should also be culturally and linguistically appropriate, focusing on the risks associated with waste handling and the importance of safety measures. Improving access to healthcare services for regular health checks and vaccinations is crucial, as is engaging waste handlers and their communities in designing and implementing health and safety protocols to ensure practicality and effectiveness.

To build on the findings of this review, future research should focus on longitudinal studies to track the effectiveness of implemented safety interventions over time. Additionally, more detailed studies that investigate the direct correlation between specific types of PPE usage and the incidence of particular injuries could provide clearer guidance for policy and practice improvements.

This systematic review and meta‐analysis has several limitations. First, most included studies used cross‐sectional designs, which preclude establishing causal relationships between occupational exposures and health outcomes among biomedical waste handlers. Second, reliance on self‐reported data for injuries and PPE use may have introduced reporting bias, including underreporting or overreporting due to stigma, recall error, or fear of workplace repercussions. Third, the geographic distribution of studies was limited, with most conducted in South Asia and Sub‐Saharan Africa, potentially restricting the generalizability of findings to other low‐resource settings. Substantial heterogeneity was observed across studies, with *I*² values exceeding 90% for several outcomes. This variability likely reflects differences in operational conditions, resource availability, safety protocols, and reporting practices across settings, complicating the generalization of pooled estimates. Subgroup analyses by region and publication year were limited by small sample sizes, and meta‐regression was not feasible.

Additional limitations include the restricted availability of high‐quality studies from diverse low‐resource countries, limited granularity regarding PPE types and waste management practices, and insufficient assessment of economic factors affecting the implementation of safety protocols. Small sample sizes in some studies may have reduced the precision of prevalence estimates, and methodological shortcomings in representativeness and outcome ascertainment may have led to underestimation of the true burden of occupational hazards.

Taken together, these limitations suggest that the pooled prevalence estimates should be interpreted with caution. Future research using longitudinal designs, more representative samples, and detailed assessment of PPE use and safety practices across varied low‐resource contexts is warranted to strengthen the evidence base and guide effective policy and practice improvements in biomedical waste management.

## 5 Conclusion

This systematic review and meta‐analysis demonstrates that biomedical waste handlers in low‐resource settings face substantial occupational risks, with a high prevalence of needlestick and sharp injuries, as well as elevated rates of hepatitis B and C infection. The use of essential protective measures, including PPE and hepatitis B vaccination, remains critically low, and training in biomedical waste management is inconsistent.

## 6 Recommendations

To mitigate these risks, policymakers and healthcare administrators should prioritize the provision of comprehensive PPE, ensure mandatory hepatitis B vaccination for all waste handlers, and implement regular, culturally appropriate training programs. Strengthening regulatory frameworks and monitoring compliance can further enhance workplace safety and reduce the occupational health burden in this vulnerable group.

## Author Contributions

**Prakasini Satapathy:** conceptualization, writing – review and editing. **Abhay M. Gaidhane:** methodology, writing – original draft, visualization, project administration. **Nasir Vadia:** software, writing – review and editing, conceptualization. **Soumya V. Menon:** methodology. **Kattela Chennakesavulu:** conceptualization, writing – review and editing. **Rajashree Panigrahi:** conceptualization, writing – review and editing. **Sanjit Sah:** methodology, conceptualization, writing – review and editing. **Kajal Samantaray:** project administration, resources. **S. Govinda Rao:** software, data curation, writing – original draft, conceptualization. **Khang Wen Goh:** data curation, writing – review and editing, writing – original draft. **Rachana Mehta:** conceptualization, writing – review and editing. **Muhammed Shabil:** writing – review and editing, project administration, conceptualization, supervision, visualization. **Mahendra Singh:** data curation, project administration, conceptualization, writing – original draft, validation, methodology, supervision. **Edward Mawejje:** project administration, methodology. **Ganesh Bushi:** methodology, writing – original draft, data curation, resources, project administration, formal analysis, investigation.

## Funding

The authors received no specific funding for this work.

## Ethics Statement

The authors have nothing to report.

## Conflicts of Interest

The authors declare no conflicts of interest.

## Transparency Statement

The corresponding authors, Rachana Mehta and Edward Mawejje affirm that this manuscript is an honest, accurate, and transparent account of the study being reported; that no important aspects of the study have been omitted; and that any discrepancies from the study as planned (and, if relevant, registered) have been explained.

## Supporting information


**Figure S1:** Summary results of the quality assessment. **Figure S2:** Sensitivity analysis for prevalence of needle and sharp injury among BMW handlers. **Figure S3:** Sensitivity analysis for prevalence of Hepatitis B & C infection among BMW handlers. **Table S1:** PRISMA 2020 Checklist. **Table S2:** Search strategy. **Table S3:** Modified Newcastle ‐ Ottawa scale.

## Data Availability

All data generated or analyzed during this study are included in this published article (and its Supplementary information files).
